# The Significance of Sudden Acute Abdominal Pain

**Published:** 1897-06

**Authors:** 


### THE SIGNIFICANCE OF SUDDEN ACUTE ABDOMINAL PAIN.

The exploratory abdominal incision has taught us much about obscure points in the surgery of that region, and among other things has let in a flood of light upon the causes of the pain that is such a frequent and important feature of diseases of the abdominal viscera. If, at the present day, in the light of all this recently acquired knowledge, discouragement comes sometimes because of a mistaken diagnosis of an abdominal affection, what must have been the condition of things before the new era in surgery allowed the peritoneal cavity to be opened with impunity? The ignorance of that time seems almost like that of the dark ages; "inflammation of the bowels" was a sort of blanket diagnosis applied to a variety of affections very different in their character and indications for treatment, and diseases of the abdomen were considered entirely within the province of the physician, matters with which the surgeon had little or nothing to do.

The rapidly acquired knowledge of this subject needs constant revision, and an excellent study of the significance of sudden acute abdominal pain has lately been made in an article by Dr. Byron Robinson, who, for the purpose of learning how best to interpret this symptom, investigates first its location. He says that there are three principal localities from which the pain arises, *viz.*; the pelvis, the region of the cæcum and that of the gall bladder. As a rule, acute abdominal pain is referred by the patient to the region of the umbilicus, at least, at first, and particularly when not associated with localized tenderness, and it is this that often makes the differential diagnosis difficult at the outset in an attack, for it is only when localized pain upon pressure begins to appear that a distinction can be made among several forms of disease attended with the general symptom of pain. In addition to the pain elicited upon pressure, another valuable localizing symptom is rigidity of the abdominal muscles, a general symptom in general peritonitis, but a local one where the affected surface is limited. This rigidity is a reflex phenomenon and serves the purpose of protecting and keeping at rest the organs in which the pathological process is going on. In some cases hyperæsthesia of the skin over the affected area is to be found in addition to the muscular rigidity, but this is not to be relied upon.

The disease most frequently met with in the ileo-cæcal region as the cause of sudden acute abdominal pain is, of course, appendicitis. In the regions of the gall bladder the passage of gall stones is the frequent cause, and in the pelvis the rupture of an ectopic gestation is most likely to give rise to the pain. Besides these the affections that are common causes of the symptoms are intestinal obstruction, the passage of a renal calculus and movable kidney. The last named trouble should by no means be forgotten, although it is only lately that any significance has been attached to it as a cause of serious symptoms.

The character of acute abdominal pain is unfortunately seldom such as to aid in making a diagnosis of its origin. The difference noted is usually in the degree rather than in the nature of the pain, although in a general way a distinction is to be made between the paroxysmal and periodic pain of strangulation of the bowels and the sudden, acute, lancinating and continuous pain of a perforation of the digestive tube, the pain in this case announcing the establishment of a general peritonitis.

The want of a guide from the character of the pain gives added value to other aids which must be called in to help in the diagnosis. Mention has already been made of the location of the pain by the sensations of the patient, by the touch, by the rigidity of the muscles and by the hyperæsthesia of the skin. An additional point may be gained by applying the doctrine of probabilities and taking into account the age, sex and previous history of the patient. For instance, sudden, acute abdominal pain in an infant or young child will probably be due to an invagination of the bowel. This will be all the more probable if the intermittent, colicky nature of the pain can be detected, and the appearance of blood in the stools will make the diagnosis certain. It is to be remembered that shock is quite conspicuous in these cases and that the evidences of acute pain may be masked by it after the onset of the attack. Abdominal tumor is often wanting in this affection.

In boys or young men, sudden, acute abdominal pain will probably be due to appendicitis. In women, during the child-bearing age, the rupture of an ectopic gestation must be one of the possible causes for consideration. But, with all the aids he can bring to bear upon the diagnosis, the physician will too frequently find himself confronted by an attack of pain which he can not explain. Fortunately, in these days he has one thing to fall back upon that seldom fails to clear up the case, that is the exploratory abdominal incision. Given a case that is at once grave and obscure, and the exploratory incision is imperatively demanded, *provided it can be made by an expert.* Operation by the inexperienced man is little, if any, to be preferred to expectant medical treatment, for although the danger from the operation, *per se*, when made by a man of moderate ability, is undoubtedly less than the danger from the disease, it must be remembered that the condition present is often one that can be detected only by an eye and touch that have had long training in the work, and that the necessary operation can be successful only in hands skilled by long dractice.—*Northwestern Lancet.*

